# Supplementary material for: Chromothripsis during telomere crisis is independent of NHEJ, and consistent with a replicative origin
Source: Genome Res. 2019 May;29(5):737–49. doi: 10.1101/gr.240705.118 (PMC6499312; doi:10.1101/gr.240705.118)
Supplement: Supplemental Material [file supp_gr.240705.118_Supplemental_file_1.zip › contigs/annotated_contigs/DB37/contig.2.DB37_length_267_mean_cov_24.2471910112.docx]

**DB37_length_267_mean_cov_24.2471910112**

```
AAGC|AACAAAGAAACCAAAAAAGAAACAAAAGAAAAA|AAAAA|AAAAAAAAAACAAGAAAGCAAGAAAGCACGAAAGCAAGCAAGCA
     >chr8:106206584-106206617 + E=2e-02 p=0e+00  >chrUn_gl000220:146355-146565 - E=6e
AGCAAGAAAGCAAGCAAGAAAGAAACGAAAGAAAGAAAGAAAGAAGAAAACGGGAAAGCAAGAAAGCACGAAAGCAAGCAAGCGAGCGA
-75
GAGAGAGAGAGAGAGAGAGAGAGAGGCTGGGCGCGGTGGCTCACGCCTGTCATCCCAGCA|GTGTGGGAGGGTGAGGAAGGCGGAGGAC

ATGA
```
